# Is *HSPG2* a modifier gene for Marfan syndrome?

**DOI:** 10.1038/s41431-020-0666-0

**Published:** 2020-06-08

**Authors:** Isabela Gerdes Gyuricza, Rodrigo Barbosa de Souza, Luis Ernesto Farinha-Arcieri, Gustavo Ribeiro Fernandes, Lygia Veiga Pereira

**Affiliations:** National Laboratory for Embryonic Stem Cells (LaNCE), Department of Genetics and Evolutionary Biology, Biosciences Institute, University of São Paulo, São Paulo, SP 05508-900 Brazil

**Keywords:** Gene expression, Animal breeding

## Abstract

Marfan syndrome (MFS) is a connective tissue disease caused by variants in the *FBN1* gene. Nevertheless, other genes influence the manifestations of the disease, characterized by high clinical variability even within families. We mapped modifier loci for cardiovascular and skeletal manifestations in the mg∆^loxPneo^ mouse model for MFS and the synthenic loci in the human genome. Corroborating our findings, one of those loci was identified also as a modifier locus in MFS patients. Here, we investigate the *HSPG2* gene, located in this region, as a candidate modifier gene for MFS. We show a correlation between *Fbn1* and *Hspg2* expression in spinal column and aorta in non-isogenic mg∆^loxPneo^ mice. Moreover, we show that mice with severe phenotypes present lower expression of *Hspg2* than those mildly affected. Thus, we propose that *HSPG2* is a strong candidate modifier gene for MFS and its role in modulating disease severity should be investigated in patients.

## Introduction

Marfan syndrome (MFS—MIM# 154700) is an autosomal dominant disorder of the connective tissue with high clinical variability both between and within families [1]. It is caused by variants in *FBN1* gene encoding fibrilin-1, the major component of microfibrils [2]. Microfibrils are present in several tissues, which makes MFS a pleiotropic disease affecting mostly the ocular, cardiovascular and musculoskeletal systems [3].

Previous works have suggested that variations in *FBN1* expression caused by polymorphisms in the gene could play a role as modifier of disease severity [4, 5]. However, giving the poor genotype–phenotype correlations and the large intrafamilial clinical variability of the disease, recent works focused on understanding how variants in other genes influence MFS phenotypes [6–8].

The effect of genetic background on phenotypic variability in MFS was demonstrated in mice by our group [9]. We showed that mg∆^loxPneo^ mice in the 129/Sv (129) isogenic background presented earlier age of onset of the disease when compared with those in the C57BL/6 (B6) background. Subsequently, we identified loci modulating the phenotypic variability in B6/129 mixed mg∆^loxPneo^ mice [8]. One locus on chromosome 4 was associated with variability of the cardiovascular phenotype [8].

One of the candidate genes we identified in this region was *Hspg2*, which encodes perlecan, a heparan-sulfate proteoglycan. Variants in *Hspg2* are associated with Schwartz–Jampel Syndrome (SJS1; MIM# 255800), an autosomal recessive disease characterized by skeletal manifestations. Knockout mice for *Hspg2* presented severe skeletal abnormalities and died around birth due to heart arrest, showing that *Hspg2* plays a role in the formation of the skeletal and cardiac system [10]. Interestingly, in our mapping study the *Hspg2* locus presented an almost suggestive association with the skeletal phenotype, indicating that it may modulate both cardiovascular and skeletal phenotypes of MFS [8].

Biochemical studies show that perlecan is also involved in maintenance of vascular homeostasis by its interaction with several extracellular matrix (ECM) components, including fibrillin-1 [11, 12]. This interaction is essential for positioning fibrilin-1 multimeres in the pericellular space and, consequently, for the assembly of microfibrils [12–14].

Given these findings, we propose that *Hspg2* is a strong candidate modifier gene for MFS. Here, we used the MFS mouse model mg∆^loxPneo^ on a mixed background (B6/129) to compare *Hspg2* expression between mild and severely affected mice. We show a correlation between *Fbn1* and *Hspg2* expression, and an association between lower *Hspg2* expression and more severe vascular and skeletal phenotypes, corroborating our hypothesis of *Hspg2* as a modifier gene of MFS.

## Material and methods

### B6/129mg∆^loxPneo^ mice tissues collection and phenotyping

B6/129mg∆^loxPneo^ mice were generated as previously described [8], and the F2 generation was analyzed at 3 months of age. The project was approved by the Ethics Committee for Animal Experimentation of the Institute of Biosciences, University of São Paulo.

Full body digital radiographic images of euthanatized animals were obtained using In Vivo Imaging System FX PRO (Bruker, Germany). Hiperkyphosis was quantified using the kyphosis index (KI) as described [15, 16]. We selected ten mice (five males and five females) with the lowest ten mice (five males and five females) with the highest KI values as the severe and mild group, respectively. Thoracic spinal column fragments were collected for RNA extraction.

Descending thoracic aorta fragments were collected from each mouse for RNA extraction and histology. For each animal, three transversal slices of the aorta were analyzed for elastic fibers fragmentation by optical microscopy, where number of fragmentations (*N*) was counted (Supplementary Fig. 1B). Elastic fibers integrity index (EFI) was calculated as following:

$$\rm{EFI} = \rm{Mean}\left( {\frac{1}{{N + 1}}} \right).$$

Mice with an EFI in the highest quartile were classified as mild (five males and 4 females) and those with an EFI in the lowest quartile as severe (four males and three females). Transversal sections of descending thoracic aorta were also screened for aneurysms. Mice with saccular aneurysms (five males; two females) were considered as a separated group.

### RNA extraction and gene expression assay

Spinal column and thoracic aorta fragments were macerated in liquid nitrogen in Trizol® reagent (ThermoFisher). RNA was isolated by RNeasy MiniSpin using RNeasy MiniKit (Qiagen). Complementar DNA (cDNA) was obtained from 500 ng of total RNA using SuperScript™ III Reverse Transcriptase (Thermofisher). For *Fbn1* and *Hspg2* expression analysis we used TaqMan® Gene Expression Assay (Thermofisher—Mm01334119_m1 and Mm01181173_g1, respectively). The gene *Actb* (TaqMan® Gene Expression Assay—Mm00607939_s1) was used as endogenous control to calculate fold change based on the 2^−[ΔΔCt]^ method [17].

### Statistical analysis

Wilcoxon nonparametric test was used for statistical analysis of comparative gene expression. Pearson correlation test was used for gene expression correlation analysis. Expression values in the aorta were adjusted for sex effects by linear model. Tests with *p* value < 0.05 were considered statistically significant. Computation was performed using R software (version 3.6.1). All data are available in the Supplementary data file.

## Results

### *Hspg2* expression and phenotypes severity

Heterozygous mg∆^loxPneo^ (F2 B6/129) animals were phenotyped for skeletal and cardiovascular systems and separated in groups according to severity (Supplementary Fig. 1A/B). Mice with aneurysm had EFI similar to those in the severe group (Supplementary Fig. 1B).

**Fig. 1:**
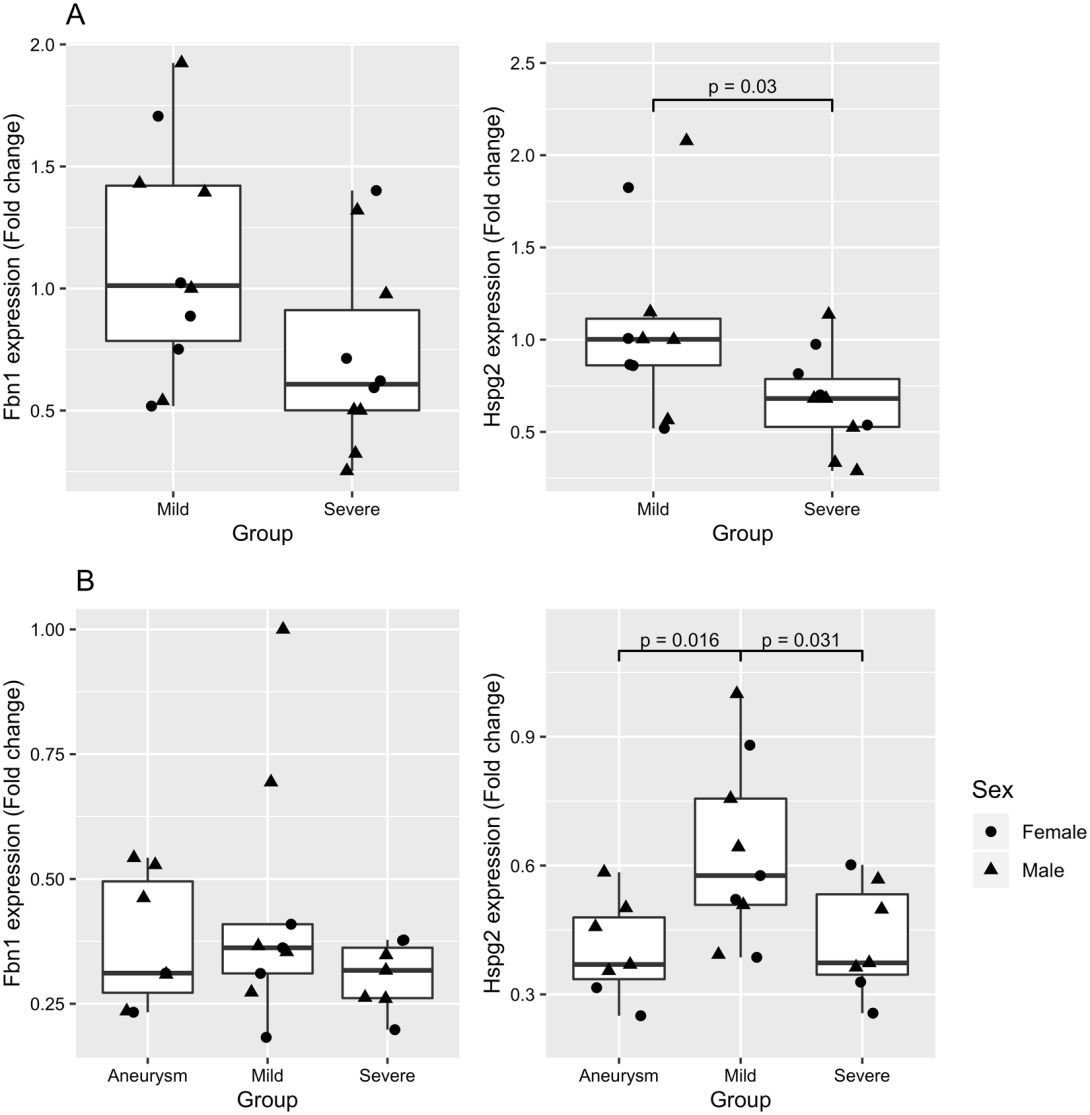
Expression of *Hspg2* and *Fbn1* in mg∆^loxPneo^ mice. Box plots with expression of *Fbn1* (left) and *Hspg2* (right) in animals with mild and severe phenotypes in **a** spinal column; and **b** aorta.

Expression of *Fbn1* and *Hspg2* was quantified in aorta and spinal column (Fig. 1). No difference of *Fbn1* expression in the spinal column and aorta was observed between severe and mild mice for each phenotype (Fig. 1). In contrast, mice with severe hyperkyphosis had lower expression of *Hspg2* in comparison with the mild group (*p* < 0.05) (Fig. 1a). Similarly, expression of *Hspg2* in aorta was lower in the severely affected and the aneurysm group when compared with mildly affected animals (*p* < 0.05) (Fig. 1b). Similar results were obtained when correcting for differences in sex distribution among groups (Supplementary Fig. 1C). No differences in *Hsgp2* and *Fbn1* expression between the different isogenic wild type and mutant mice were detected (data not shown).

### Correlation between *Fbn1* and *Hspg2* expression

**Fig. 2:**
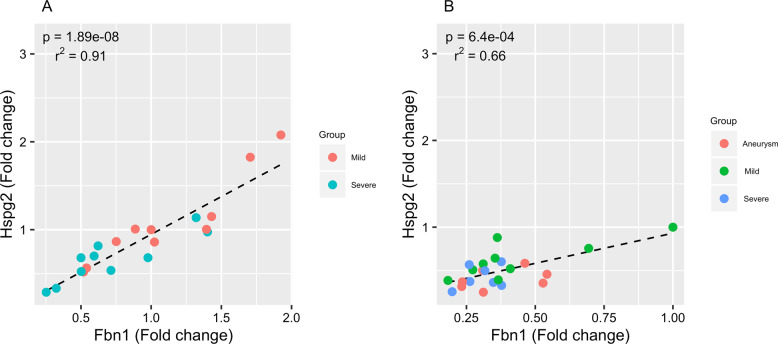
Correlation between *Fbn1* and *Hspg2* expression in mg∆^loxPneo^ mice. Scatter plots showing positive correlation between *Fbn1* and *Hspg2* expression in **a** spinal column from mg∆^loxPneo^ mice with severe and mild skeletal phenotype; **b** in aorta from the three mg∆^loxPneo^ groups of vascular phenotype.

Although we did not find any differences in *Fbn1* expression between groups in the two phenotypes analyzed, we tested for correlation between *Fbn1* and *Hspg2* expression which could suggest co-function of the corresponding proteins. We observed a significant positive correlation between *Fbn1* and *Hspg2* expression on both spinal column and aorta in mgΔ^loxPneo^ mice (*p* < 0.01 | *r*^2^ = 0.66 and *p* < 0.01 | *r*^2^ = 0.91, respectively) (Fig. 2).

## Discussion

The mg∆^loxPneo^ mouse model of the clinical variability of MFS allowed us to identify the Awtq1 locus in mouse chromosome 4/human chromosome 1 as a modifier of the cardiovascular phenotype. Within that locus we highlighted *Hspg2* as a candidate modifier gene based on its involvement in skeletal and cardiovascular function, and its direct interaction with fibrillin-1 [10, 12, 18].

More recently, a study with 1070 MFS patients identified a smaller modifier locus for the cardiovascular phenotype named gMod-M1 which overlaps with Awtq1 [7]. The only candidate gene identified within that locus was *ECE1*, highly expressed in the aortic wall and involved in regulation of endothelial-to-mesenchymal transition [7]. However, the region also contains the *HSPG2* gene which, interestingly, has a 2.5-fold higher expression in aorta than *ECE1* (227.2 TPM vs. 93.88 TPM, *n* = 432; GTEx Portal). Thus, by the expression and function criteria, *HSPG2* should also be considered a candidate modifier gene.

Our expression data from mg∆^loxPneo^ mice show a positive correlation between *Hspg2* and *Fbn1*, strengthening the hypothesis of co-function of the corresponding proteins [12–14, 19]. Moreover, we showed that lower expression of *Hspg2* is associated with more severe hyperkyphosis, and with lower integrity of elastic fibers and presence of aneurysms. Thus, although the *Hspg2*/*HSPG2* locus has been originally identified as a modifier of the cardiovascular phenotype, our findings indicate that *Hspg2* expression may influence the severity of both skeletal and vascular phenotypes in MFS mice.

The identification of modifier genes of monogenic diseases gives clues about the molecular mechanism of pathogenesis and novel therapeutic strategies and contributes to the prediction of disease severity. Here, we build a case for *HSPG2* as a modifier gene for MFS by reviewing the literature and presenting corroborating evidence in our mouse model of MFS clinical variability. We propose that the role of *HSPG2* in modulating the severity of skeletal and cardiovascular manifestations should be investigated in large cohorts of MFS patients.

## Supplementary information

Supplemental text

Supplemental Figure 1

Supplemental Figure 1 legend

Supplemental Data table
