# Supplementary material for: Is HSPG2 a modifier gene for Marfan syndrome?
Source: Eur J Hum Genet. 2020 Jun 8;28(9):1292–6. doi: 10.1038/s41431-020-0666-0 (PMC7608216; doi:10.1038/s41431-020-0666-0)
Supplement: Supplementary file 1 — Supplemental text [file 41431_2020_666_MOESM1_ESM.docx]

**Supplemental material**

Supplemental Figure 1 A and B show phenotyping methods and results. A) The x-ray picture shows the skeletal classification method by quantification of kyphosis index (upper panel) and the box plots (lower panel) show the difference in KI for mild and severe groups. B) The histology aortic sections (upper panel) show methods used for vascular phenotyping: presence/absence of aneurysm (right), and computation of elastic fibers integrity index (EFI) based on elastic fibers fragmentation (left). The box plots (lower panel) show that both severe and aneurysm groups presented reduced EFI when compared to the mild group. Supplemental Figure 1C shows box plots for the expression of *Hspg2* (left) and *Fbn1* (right) after correcting for differences in sex distribution among groups using linear model.

Supplemental Data consists of a datasheet with multiple tabs providing access to all the data generated in this work. We provided the measurements for vascular and skeletal phenotypes for all B6/129 mg∆^loxPneo^ mice (Phenotype_data), and gene expression data from the B6/129 mg∆^loxPneo^ mice tested, such as fold-change values for *Fbn1* and *Hspg2* (Norm_expression_data), and raw Ct values from qPCR for *Fbn1* and *Hspg2* from aorta (Raw_expression_aorta) and spinal column (Raw_expression_spinal_column).
