# Supplementary figures and images for: Is HSPG2 a modifier gene for Marfan syndrome?
Source: Eur J Hum Genet. 2020 Jun 8;28(9):1292–6. doi: 10.1038/s41431-020-0666-0 (PMC7608216; doi:10.1038/s41431-020-0666-0)

A

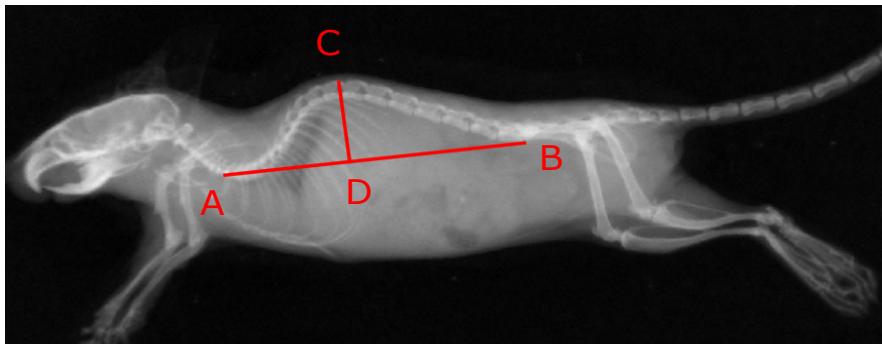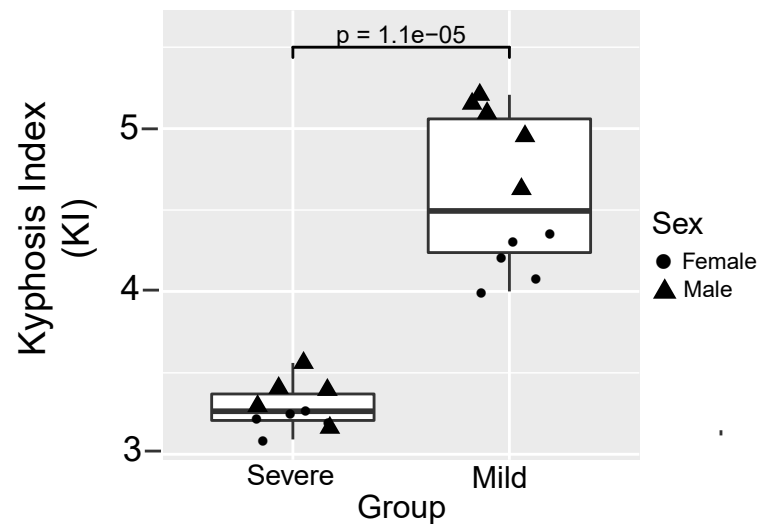

B

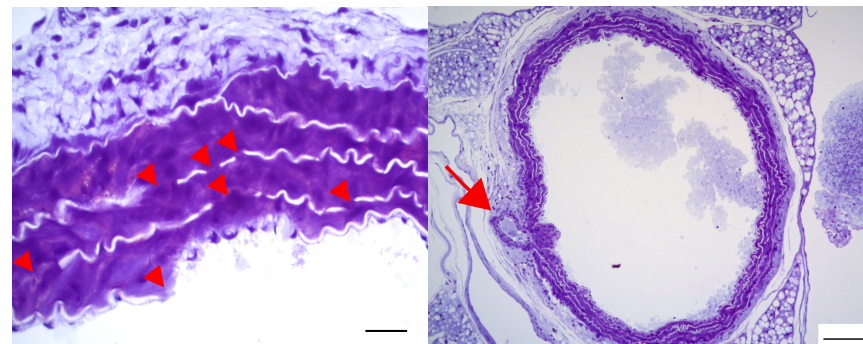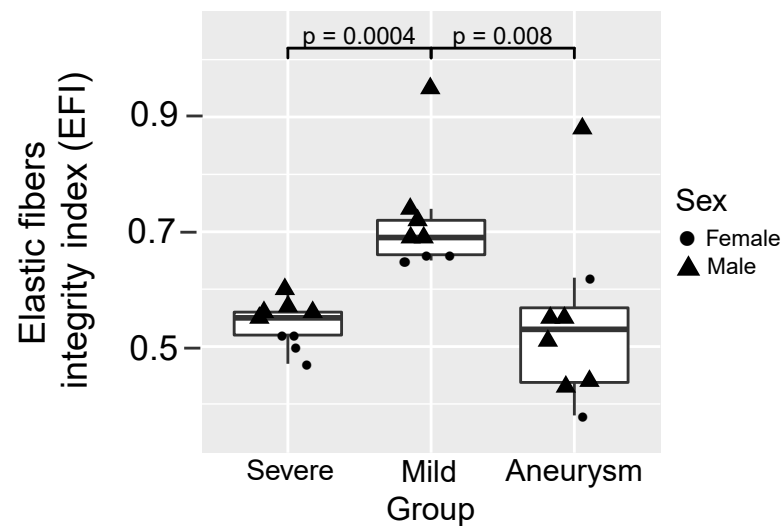

C

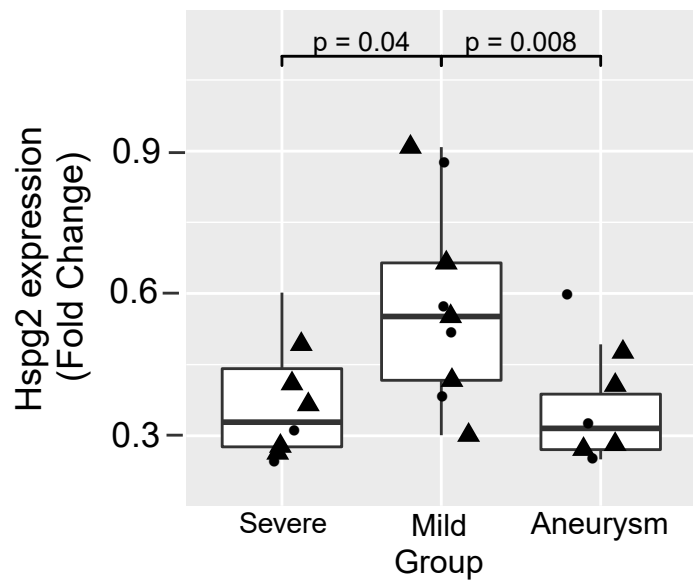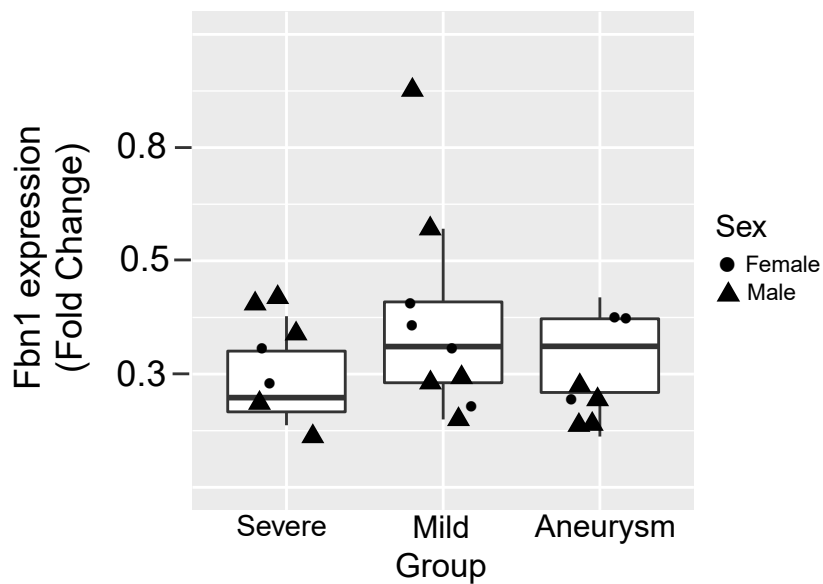

Supplement: Supplementary file 2 — Supplemental Figure 1 [file 41431_2020_666_MOESM2_ESM.pdf]
