# Supplementary material for: Is HSPG2 a modifier gene for Marfan syndrome?
Source: Eur J Hum Genet. 2020 Jun 8;28(9):1292–6. doi: 10.1038/s41431-020-0666-0 (PMC7608216; doi:10.1038/s41431-020-0666-0)
Supplement: Supplementary file 3 — Supplemental Figure 1 legend [file 41431_2020_666_MOESM3_ESM.docx]

**Supplemental Figure 1.** (A) Skeletal phenotype. Left: x-ray showing lines used for the calculation of KI (KI=|AB|/|CD|); right: box plots with values for KI for animals from the severe and mild groups. (B) Vascular phenotypes. Right: histology of cross sections of the aorta in mg∆^loxPneo^ animals. Red arrowheads show sites of elastic fiber fragmentation (scale bar 10 µm) and red arrow points to aneurysm (scale bar 50µm); left: Box plots with values for elastic fibers integrity index (EFI) for animals from the severe, mild and aneurysm groups; (C) Gene expression after sex adjustment**.** Box plots with aortic expression of *Hspg2* (left) and *Fbn1* (right) for the mild, severe and aneurysm groups.
